# Whose conservation, revisited: how a focus on people–nature relationships spotlights new directions for conservation science

**DOI:** 10.1098/rstb.2023.0320

**Published:** 2025-01-09

**Authors:** Belinda Reyers, Elena M. Bennett

**Affiliations:** ^1^ Centre for Environmental Studies, University of Pretoria, Pretoria 0028, South Africa; ^2^ Beijer Institute of Ecological Economics, Royal Swedish Academy of Sciences, Stockholm, Sweden; ^3^ Department of Natural Resource Sciences and Bieler School of Environment, McGill University, Montreal H9X 3V9, Canada

**Keywords:** transformative change, relational values, social-ecological systems, sustainable development, Anthropocene, biodiversity

## Abstract

Georgina Mace introduced a compelling perspective on the major shifts in conservation science’s framing and purpose from 1960 to 2010. A decade ago, she proposed that the conservation community had begun to move into a new framing of ‘people and nature’ based on changes in perspectives on the relationships between people and nature and new interdisciplinary concepts and methods used in conservation. Progress in using this frame is clear as ‘two-way dynamic relationships between people and nature’ have since taken centre stage in science, practice and policy. Now, responding to concerns raised that current approaches to conservation are still not meeting the scale and complexity of the challenges of the Anthropocene, we explore a newly emerging framing of ‘people with nature’—an inextricably intertwined perspective on people–nature relationships. This framing builds on Mace’s recognition of interconnections and change, as well as new directions offered by conservation’s recent transdisciplinary engagements, to go beyond the notion of two-way flows connecting people and nature to emphasize the relationships and inseparability of ‘people with nature’. This emerging framing suggests new directions for conservation science and practice to make visible, improve and reimagine degraded people–nature relationships needed to bend the curve of biodiversity loss.

This article is part of the discussion meeting issue ‘Bending the curve towards nature recovery: building on Georgina Mace's legacy for a biodiverse future’.

## 1. Introduction

**Figure 1.**
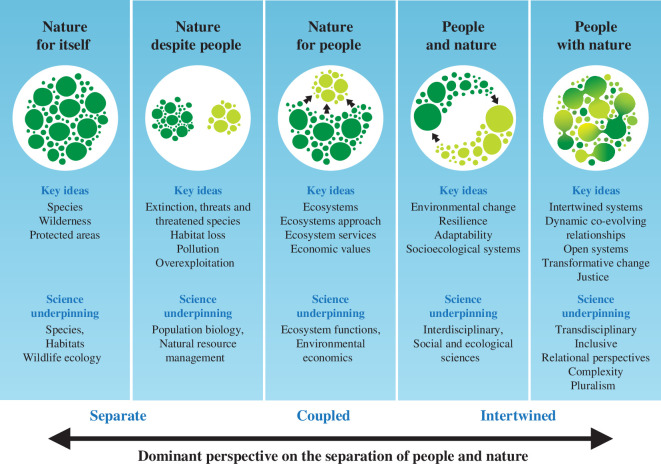
Major shifts in conservation’s framing and purpose, as well as key ideas and the science underpinning each framing introduced by Georgina Mace [1]; now updated to include a newly emergent framing of ‘people with nature’ and its novel ideas and science requirements. This new framing helps to clarify the different perspectives of each framing on the separation and separability of people and nature, moving over time to focus more on the couplings and linkages connecting people and nature. In the newly emerging framing, the inseparability of people and nature is highlighted, with a focus on the relationships that make ‘people with nature’ intertwined and inseparable. Graphics created by Jive Media Africa.

A decade ago, the late Professor Georgina Mace introduced a compelling perspective on the evolution of global conservation science, suggesting that there had been four major shifts in its framing and purpose since the 1960s [1] (figure 1). She highlighted how conservation in the 1960s tended to prioritize wilderness and exclude people, a framing that she labelled ‘nature for itself’. In the 1970s and 1980s, this shifted to a ‘nature despite people’ framing as awareness of the environmental crisis and the impacts of human actions on nature grew. A decade on, a focus on the benefits that nature provides to people and an increased appreciation of the costs of environmental degradation to human societies and economies moved conservation into a ‘nature for people’ framing. Mace [1] highlighted that these shifts were underpinned by changes in the dominant perspective on the relationships between people and nature. In her paper, which has been a touchstone for contemporary conservation science, she proposed that the global conservation community had just begun to move into a new framing, which she called ‘people and nature’. In this framing, interdisciplinary methods underpin a conservation science that accepts social and ecological interdependence and recognizes change as a constant.

The past decade has borne witness to much progress using this framing, evidenced by an increased focus of science, policy and practice on the "two way dynamic relationships between people and nature" [1]. Key achievements helped along by this more integrated framing include: a deeper and broader engagement of the social sciences in conservation science [2]; more interconnected conceptual frameworks underpinning global policy and science-policy processes, e.g. the Intergovernmental Science-Policy Platform on Biodiversity and Ecosystem Services [3], the UN 2030 Agenda and its 17 interconnected Sustainable Development Goals [4]; and a wider appreciation of the importance of both people and nature in conservation and sustainable development practice [5,6].

As Mace [1] points out, the ‘tendency to treat people and nature as separate units in discourse and analysis’ has become far less prevalent in conservation science and policy, which has facilitated a broader engagement of disciplines, stakeholders and knowledge systems in conservation science. This broader engagement has, in turn, led to new areas of interdisciplinary research and action, more integrated global policy frameworks, and a more open and dynamic conservation science better suited to the dynamic and globalized nature of the Anthropocene [7,8].

## 2. Bending the curve of the Anthropocene’s conservation and sustainable development trends

Mace [1] highlighted that many of the changes in conservation science framings over time were motivated by a concern that conservation efforts were failing, as extinction rates and other metrics of biodiversity loss continued to increase despite global efforts to address these serious problems. Her proposal of the ‘people and nature’ framing helped by highlighting the cultural structures and institutions important to the long-term sustainability and resilience of the connections between people and nature. Despite progress emerging from the ‘people and nature’ frame, recent global assessments and analyses highlight that current approaches to conservation are still not meeting the scale and complexity of the challenges posed by the Anthropocene, as evidenced by the continuing and even accelerating pervasive declines in biodiversity and the continued large-scale alteration of multiple ecosystem and earth system processes [9,10].

At the same time, the world is witness to the first ever reported declines in human development, with the Human Development Index dropping in 2020–2022 to levels last seen in 2015 [11]. While it is possible to link some of these declines to the impacts of the COVID-19 pandemic, other troubling changes, such as rising and globalizing inequalities [12], increases in human insecurity and perceptions of insecurity [13] and widespread polarization of societies [11], predate the pandemic. These disturbing social and ecological trends, and their increasingly complex interconnections, are a hallmark of the Anthropocene, highlighting the new challenges that this era presents for biodiversity conservation as well as broader sustainable development efforts [14,15]. Recent research demonstrates the globally interconnected and dynamic nature of social and environmental trends and crises in what some refer to as a 'polycrisis' [16]. The prospect of a polycrisis raises questions and concerns about the potential for the Anthropocene to become a trap of maladaptive or undesirable trajectories amplifying one another in a series of feedbacks and persistent crises, resulting in the continuing degradation of both the environment and human wellbeing [9,13,16–18].

That important conservation *and* sustainable development metrics are on the decline points to the failure of both to take into account the globally intertwined, highly unequal and dynamic social–ecological systems of the Anthropocene [13,15]. As Mace [1] began to highlight, the causes of Anthropocene challenges lie within the economic, political, cultural and social structures and systems–along with their dynamics and power asymmetries–that all underlie the current polycrisis [19,20]. Such complex social–ecological dynamics and structures reinforce and link environmental pressures and social imbalances across scales of time and space, requiring new knowledge, approaches, capacities and collaborations in conservation to allow us to understand and correct these unsustainable trajectories of the Anthropocene [11].

Building on the progress made using Mace’s [1] ‘people and nature’ framing and its recognition of interconnections and change, we propose that conservation science continues to evolve, responding to new understanding and new challenges, now advancing towards a new emerging framing that we label ‘people *with* nature’—an inextricably intertwined perspective on people–nature relationships (figure 1). This emerging framing offers new and diverse perspectives and approaches, as well as capacities to better understand and engage with complex and dynamic people–nature relationships of the Anthropocene. These relationships are at the core of Mace’s [1] evolution of conservation framings and, we propose, also at the heart of the problem of bending the curve on biodiversity loss and other sustainable development trends of the Anthropocene [21–23].

## 3. ‘People with nature’: emphasizing relationships that make up the whole

This emerging conservation framing of ‘people with nature’, which we aim to explore here, seeks to clarify that conservation’s depictions of the interactions and relationships between people and nature vary in terms of the degree to which the human and natural systems are viewed as loosely coupled, embedded or part of a single integrated system [24,25] (figure 1). The ‘people *and* nature’ perspective of two-way material and energy flows connecting people and nature sits on the more loosely coupled side of this spectrum. However, the emerging framing of ‘people with nature’ recognizes that the boundaries we use to separate people from nature when depicting these flows in conservation science are artificial, and therefore arbitrary [6,26]. It does away with the distinctions between humans and nature by no longer focusing on these entities, but rather on the social–ecological relationships that make up an integrated social–ecological system [24,27,28]. The new framing follows from recent advances made on many fronts, including the study of complex adaptive systems [29,30], new interdisciplinary engagements of conservation with the social sciences [31], new linkages with relational approaches in sustainability science [32] and the recent move to decolonize conservation science and mobilize global south scholarship, Indigenous knowledge and local knowledge systems into conservation research and practice [6,26,33,34]. These advances have highlighted the need to move beyond reductionist tendencies to break systems into social and ecological components for study (and then from these separate understandings to try to reconstruct the ‘whole’). Rather, they call for approaches that emphasize the relationships that connect the parts and make up the intertwined whole. Such relational approaches, with their focus on interconnectedness, dynamic relationships and processes, appear better suited for studying and engaging in the hyperconnected and ever-changing contexts of contemporary conservation science [32].

**Figure 2.**
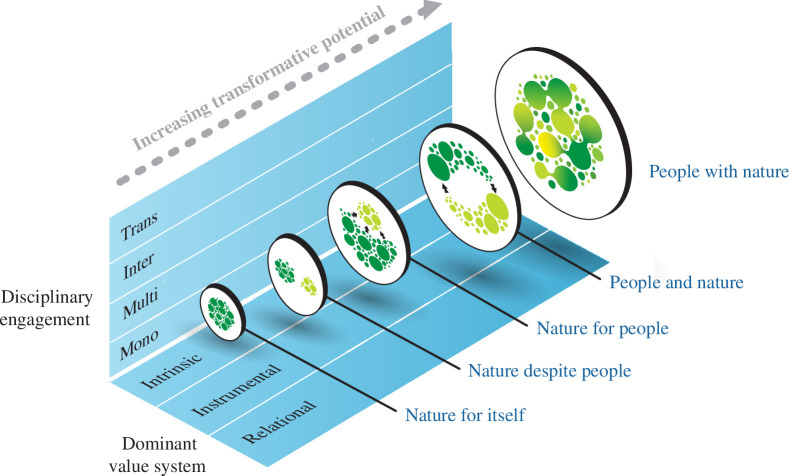
The framings used in conservation science differ in terms of the dominant value systems that are emphasized in each framing, moving from a focus on intrinsic and subsequently instrumental values and more recently to relational values in the newly emerging framing of ‘people with nature’. At the same time, conservation science has shifted in its depth and breadth of engagement with disciplines and other knowledge systems, moving from a focus on disciplinary sciences to multi- and inter-disciplinary interactions and more recently in the ‘people with nature’ perspective fostering transdisciplinary engagements beyond academia that are more inclusive of diverse knowledge systems. As conservation science perspectives shift along these two axes, they evolve into a science and practice that accept, embrace and even promote change, navigating uncertainty and fostering learning by promoting the processes of transformative (system-wide) change that are needed to reveal, restore and reimagine people–nature relationships. Graphics created by Jive Media Africa.

By foregrounding the dynamic relationships connecting people and nature, many of the sectoral divisions and tensions that emerge from treating conservation and human development as separable and standalone can be overcome. Instead of focusing on linear trade-offs or synergies between outcomes for nature and outcomes for people, the ‘people with nature’ framing focuses on the nature and quality of relationships between the two, which offers important opportunities for a more dynamic and holistic analysis [35]. The new framing moves conservation science away from instrumentalist values and assumptions of competition between conservation and development to a more collaborative and relational problem framing (figure 2). Such a framing is better able to consider biodiversity loss, poverty and inequality as inextricably interlinked, and as emergent outcomes of system structures and dynamics rather than as linear causes or effects of one another [15]. This, in turn, moves away from older tensions in the conservation community between intrinsic and instrumental values, and towards a more inclusive arena of people–nature relationships and relational values [22,36] (figure 2). Thus, the new framing suggests that problems of conservation or issues of development can *only* be truly addressed in concert with one another; there is no possibility to address one at the expense of the other because there is no ‘one’ or ‘the other’—there is only the co-evolving relationship of people with nature, with each shaping and being shaped by the other [25,37].

Such a relational framing of ‘people with nature’ means that conservation science converges with, and benefits from, recent advances in other domains that highlight the diverse, dynamic and relational nature of human wellbeing and its relationships with—and values assigned to—nature (e.g. [22,38–40]). These studies find that human wellbeing is more than just material or individual, but rather that wellbeing is relational and dynamic because it is shaped by the ever-changing interplay of people with nature and by diverse value systems across scales. Global South scholarship on the topic of people–nature relationships offers a more inclusive and interconnected representation of wellbeing and its links to nature, including spiritual, collective and distributive dimensions (intra-generational and inter-generational) that are inextricable from the environment (e.g. [6]). Furthermore, by adopting a relational perspective on people–nature interactions, conservation science aligns with and mobilizes other, often marginalized, local and Indigenous knowledge systems that have not been constrained by reductionist and instrumentalist perspectives and thus offer novel insights, theories and methods [26,41,42] for conservation science to collaboratively move into a more relational ‘people with nature’ framing.

## 4. New relational approaches and perspectives for bending the curve

Mace [1] pointed to the need for a more interdisciplinary conservation science to be able to integrate social and natural sciences in the ‘people and nature’ framing. In this emerging framing, conservation science goes even further, becoming a transdisciplinary endeavour that engages with diverse relational knowledge systems, values and practices (figure 2). As Mace [1] made clear, the emergence of new framings does not entirely replace previous framings; multiple framings can co-exist. But what is emerging in conservation science is an awareness of these diverse perspectives, an ability to recognize the contribution of each different framing and the skills to work with diverse framings while advancing the transdisciplinary innovations offered by the new emerging framing of ‘people with nature’. These innovations include robust methods and practices founded on relational and complexity-oriented approaches [43], increasing collaborations with the humanities in areas such as art, law and philosophy [44,45] and new transdisciplinary processes working to co-produce, mobilize and interweave the diverse knowledge systems needed to bend the curve [34,46,47].

A ‘people with nature’ framing in conservation science has implications for not only how conservation science is performed, but also how conservation science conceptualizes key factors such as scale and change. To date, science that has focused on people–nature relationships has typically implied that the scale of interest is single, 'local' and relatively small (e.g. a community or a park). However, more recently, as the ‘nature for people’ and ‘people and nature’ framings have become prevalent, conservation science has recognized that the relationships between people and nature exist at multiple scales, thus necessitating multi-scale studies and methods [14,48].

The increasingly complex and interconnected nature of the Anthropocene and the dominant role of external factors (e.g. trade and policy impacts) in local contexts (and *vice versa*) make clear that system boundaries are porous and in constant flux [49]. These are radically open systems [29], meaning that people–nature relationships do not only exist at a single scale, but span and connect scales in ever-changing ways with effects that ripple around the world, affecting outcomes at other scales and at distant locations [50–52]. Conservation science must thus address the complexity of people–nature relationships as neither local nor global but rather as cross-scale and dynamic. Such a re-framing shifts conservation’s theories and methods towards system and complex system approaches that can account for external factors and the political and economic structures and systems [20,52,53] that lie behind biodiversity loss and work across scales. Such a perspective is more mindful of the potential for complex cross-scale feedbacks of interventions that might affect biodiversity or sustainable development more broadly in often far distant locations [6,13,54].

There are several recent examples of conservation science’s diverse engagements with relational, transdisciplinary, cross-scale and complexity-oriented perspectives that align with the emerging framing of ‘people with nature’. However, perhaps unsurprisingly, there are few examples (to our knowledge) that bring them all together. For example, biocultural diversity and biocultural conservation are promising conceptual frameworks bringing together multiple knowledge systems, decolonizing and transdisciplinary methodologies and relational approaches for biodiversity conservation [55]. Their emphasis on cultural practices and approaches such as agrobiodiversity and cultural landscapes for biodiversity conservation have proven valuable in foregrounding the interconnections between people and nature. However, as Cocks and Wiersum [56] argue, biocultural approaches in conservation are mostly limited in application to the local scale for groups of people for whom there is a direct and obvious relationship with nature. These approaches mostly exclude the dynamic, cross-scale and complex nature of people–nature relationships—which are increasingly relevant in the Anthropocene context—and the resultant increases in the distance and disconnect between people and nature [57].

Landscape and stewardship approaches can help integrate relational approaches with complexity-oriented frameworks. For example, the landscape stewardship work of Cockburn *et al*. [58] proposes that frameworks of social–ecological systems paired with relational landscape approaches can help to incorporate multiple, dynamic and intersecting human–nature and human–human relations in research, policy and practice. Using such relational approaches, the authors are able to highlight diverse cross-scale relationships important in water conservation efforts, including between farmers, water sources, government officials and users, and the dynamic and multi-scale nature of these relationships.

These and many other novel engagements offer insights into this emerging direction and potential value of the ‘people with nature’ perspective. Diverse relational, complexity and transdisciplinary methods, capacities and applications have different disciplinary origins, areas and scales of application, value orientations and communities of practice. The ‘people with nature’ framing benefits from this diversity and pluralism in transdisciplinary collaborations and learning, drawing on these separate and emerging fields [59].

## 5. People with nature: novel collaborations for bending the curve

Georgina Mace’s ‘people and nature’ framing was instrumental in moving conservation science into a more dynamic space by acknowledging the ubiquity of change. By combining relational, complexity-oriented and transdisciplinary approaches, the ‘people *with* nature’ framing goes beyond acknowledging change to emphasize the continuously unfolding, nonlinear nature of change and the co-evolution of people–nature relationships that shape, and are shaped by, conservation and development efforts [25]. This new framing pushes conservation science away from a focus on conservation and sustainable development goals or targets as defined end points, towards process-oriented approaches that account for learning, experimentation and the need to navigate emergence and uncertainty [28,60,61]. The ubiquity of change and associated uncertainties—especially in the context of the Anthropocene, with its heightened pace and scale of change—present a fundamental challenge for conservation science and highlight the need for more dynamic perspectives and approaches. These must not only embrace the dynamic nature of social–ecological systems and their potential threshold effects and tipping points, but also confront the uncertain, unsettled and increasingly polarized social and political context in which conservation efforts take place [11,13,16].

With its focus on dynamic, cross-scale and co-evolving people–nature relationships, a ‘people with nature’ framing not only acknowledges change, but also seeks to promote it, rather than resist it. As conservation science moves into this new framing, it becomes clear that the change it seeks to promote is not an incremental improvement in conservation outcomes (e.g. protected area extent). Such incremental improvements seek to preserve the system in its current state, return it to some prior state or perhaps adapt it to social and environmental changes while retaining similar patterns and functions. The ‘people with nature’ framing instead promotes the processes of transformative change. Transformative change is defined as the system-wide reconfiguration of systems and their relationships in a way that shifts ‘authority, resource flows, power, norms, values and beliefs and the social–ecological relationships’ that create the conservation challenge in the first place [60] (figure 2). The framing thus focuses on the changes needed in the political, social and economic structures and dynamics underpinning biodiversity loss, as well as their underlying values and paradigms that are often at odds with bending the curve. It is to these deeper leverage points that recent global assessments of biodiversity loss suggest conservation efforts need to move [15].

By engaging with transformative change, this emerging framing benefits from scholarship and learning in the field of transformations. Such learning highlights the importance of people–nature relationships, emphasizes the degraded nature of these relationships and the undesirable lock-ins that now exist in them (e.g. the self-reinforcing feedbacks between industrial agricultural, biodiversity loss and climate change). Such degradation and lock-ins put the world on course for further biodiversity losses, environmental degradation and other sustainable development risks and challenges [10]. Transformative change research also introduces novel concepts and approaches such as leverage points, transformative capacities and scaling to the conservation community [21,28,36,60,62], which appear crucial in the context of the Anthropocene. Furthermore, through its engagement with transformative change, the framing of ‘people with nature’ can become more aligned with principles of inclusivity, equity, diversity and plurality, which are central to processes of transformative change [63] and have in the past been highlighted as problematic or marginal but essential to conservation science [64–67].

Working within the framing of ‘people with nature’ also opens up novel approaches, collaborations and partnerships needed to bend the curve. Through an embrace of relational, transdisciplinary, transformative and decolonial approaches, conservation science finds new collaborations and partners in social justice [68], indigenous governance innovations [69,70], youth engagement [71], food sovereignity initiatives [72] and many others also working to target the same underlying structures, systems and norms [15], but from different perspectives and for different reasons.

## 6. Conclusion

The scale and complexity of the challenges of the Anthropocene, including that of bending the curve of biodiversity loss, are daunting. In fact, when viewed from perspectives that treat nature and people as separate and separable they can appear insoluble. Such views often leave biodiversity loss in the hands of a few ‘conservationists’ trying to raise awareness and make the case for biodiversity conservation against a backdrop of the social, political and economic crises of the Anthropocene and its many competing priorities. From a ‘people *with* nature’ perspective, the goal of bending the curve of biodiversity loss has the potential to become a more collaborative goal, drawing conservation into novel collective processes, building, restoring and rewiring people–nature and people–people connections in ways that benefit nature *and* people.

Importantly, the emerging ‘people with nature’ framing will require new networks, capacities and practices in conservation science, not least of which will be the abilities to reimagine degraded people–nature relationships, reconfiguring them to set social–ecological systems onto new trajectories and the diverse competences needed to navigate uncertain futures together. Georgina Mace’s focus on people–nature relationships and her call for a more inclusive focus in conservation science are even more relevant today than they were a decade ago. The emerging framing of ‘people with nature’ that we highlight here builds on her legacy, with the potential to transform our approach to conservation and sustainable development.

## Data Availability

This article has no additional data.
